# New “missing link” genus of the colonial volvocine green algae gives insights into the evolution of oogamy

**DOI:** 10.1186/1471-2148-14-37

**Published:** 2014-03-03

**Authors:** Hisayoshi Nozaki, Toshihiro K Yamada, Fumio Takahashi, Ryo Matsuzaki, Takashi Nakada

**Affiliations:** 1 Department of Biological Sciences, Graduate School of Science, University of Tokyo, 7-3-1 Hongo, Bunkyo, Tokyo 113-0033, Japan; 2 College of Life Sciences, Ritsumeikan University, 1-1-1 Noji-higashi, Kusatsu, Shiga 525-8577, Japan; 3 Institute for Advanced Biosciences, Keio University, 246-2 Mizukami, Kakuganji, Tsuruoka, Yamagata 997-0052, Japan; 4 Systems Biology Program, Graduate School of Media and Governance, Keio University, Endo 5322, Fujisawa, Kanagawa 252-0882, Japan

## Abstract

**Background:**

The evolution of oogamy from isogamy, an important biological event, can be summarized as follows: morphologically similar gametes (isogametes) differentiated into small “male” and large “female” motile gametes during anisogamy, from which immotile female gametes (eggs) evolved. The volvocine green algae represent a model lineage to study this type of sex evolution and show two types of gametic unions: conjugation between isogametes outside the parental colonies (external fertilization during isogamy) and fertilization between small motile gametes (sperm) and large gametes (eggs) inside the female colony (internal fertilization during anisogamy and oogamy). Although recent cultural studies on volvocine algae revealed morphological diversity and molecular genetic data of sexual reproduction, an intermediate type of union between these two gametic unions has not been identified.

**Results:**

We identified a novel colonial volvocine genus, *Colemanosphaera*, which produces bundles of spindle-shaped male gametes through successive divisions of colonial cells. Obligately anisogamous conjugation between male and female motile gametes occurred outside the female colony (external fertilization during anisogamy). This new genus contains 16- or 32-celled spheroidal colonies similar to those of the volvocine genera *Yamagishiella* and *Eudorina*. However, *Colemanosphaera* can be clearly distinguished from these two genera based on its sister phylogenetic position to the enigmatic flattened colonial volvocine *Platydorina* and external fertilization during anisogamy. Two species of *Colemanosphaera* were found in a Japanese lake; these species are also distributed in European freshwaters based on a published sequence of an Austrian strain and the original description of *Pandorina charkowiensis* from Ukraine.

**Conclusions:**

Based on phylogeny and morphological data, this novel genus exhibits a missing link between *Platydorina* and the typical spheroidal colonial volvocine members such as *Pandorina* or *Yamagishiella*. Considering the external obligate anisogamy, oogamy evolution may have been preceded by the transition from external to internal fertilization during anisogamy within the volvocine green algae.

## Background

The volvocine algae from unicellular *Chlamydomonas* to multicellular *Volvox* represent an “evolutionary time machine” model lineage to study the evolution of female–male sexual dimorphism and multicellularity because they encompass the evolutionary ranges of vegetative and reproductive morphologies between these two extremes [1,2]. Based on chloroplast multigene phylogeny, a major evolutionary scenario within the lineage was resolved ranging gradually from primitive unicellular organisms with conjugating gametes of identical size (isogamy) to the advanced multicellular spheroid with differentiation of male–female sexes (oogamy) such as *Volvox*[3-5]. The colonial volvocine algae show two types of gametic union: conjugation between isogametes outside the parental colonies (external fertilization during isogamy) and fertilization between small motile male gametes (sperm) and large female gametes (eggs) inside the female colony (internal fertilization during anisogamy and oogamy). Although recent cultural studies on the volvocine algae revealed morphological diversity and molecular genetic data of sexual reproduction [6-9], an intermediate between these two gametic unions has not been demonstrated.

The colonial volvocine algae include two morphologically unique organisms: *Volvox* sect. *Volvox* with thick cytoplasmic bridges between cells and *Platydorina* with flattened vegetative colonies developing via unique morphogenesis “intercalation” [7]. However, their phylogenetic positions are ambiguous even using multiple chloroplast genes [4,5], possibly due to the lack of closely related sister lineages that may represent their ancestral morphological traits. Thus, identifying the missing links may resolve these problems. Although Coleman [10] confirmed that the *Eudorina cylindrica* strain ASW05157 (originating from Regelsbrunn, Austria [11]) is sister to *Platydorina*, its morphological details were not reported; however, the internal transcriber spacer (ITS) of nuclear ribosomal DNA (*r*DNA) of this strain is now available (AF182439).

During our field surveys of freshwater algae in Japan, two colonial volvocalean green algae were collected from a lake. Although these algae are closely related to *Platydorina* in molecular phylogeny, their vegetative colonies are spheroidal in shape, resembling those of the colonial volvocine genera *Yamagishiella* and *Eudorina* (Volvocaceae) under a light microscope [12]. Notably, this new genus showed “external fertilization” between male and female gametes. Morphology, sexual reproduction, taxonomy, and phylogenetic significance of the two species of *Colemanosphaera* gen. nov. are described in this report.

## Results and discussion

### Morphology and taxonomy

**Figure 1:**
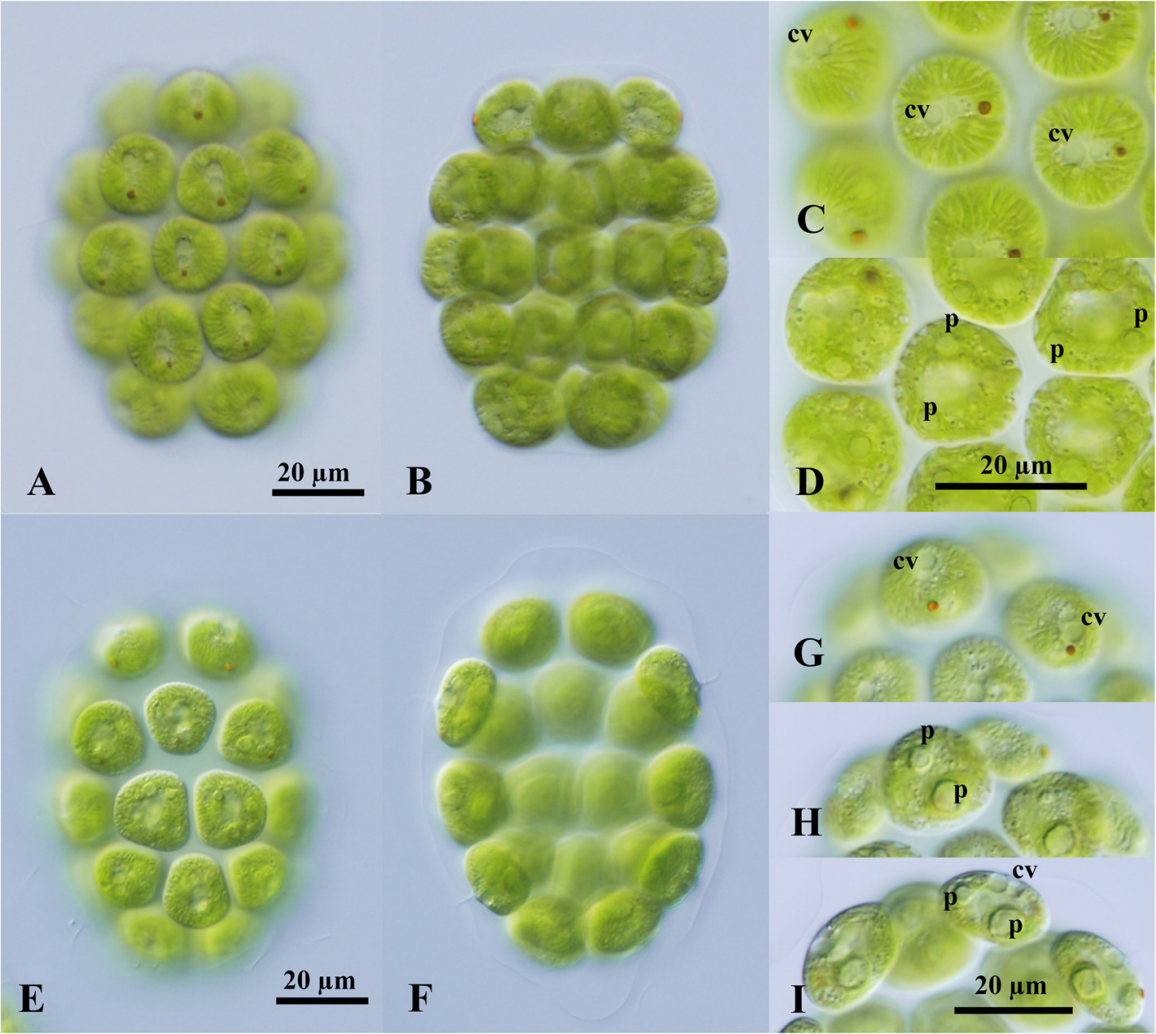
**Light microscopy of vegetative colonies of two species of *Colemanosphaera*. (A)**-**(D) ***C. charkowiensis* (Korshikov) Nozaki et al. comb. nov. 2013-0615-IC-7. **(A)**, **(B)** Two views of a 32-celled colony shown at the same magnification. **(A)** Surface view. **(B)** Optical section. **(C)**, **(D)** Two views of cells shown at the same magnification. **(C)** Surface view showing contractile vacuoles (cv) distributed in only the anterior end of cells. **(D)** Optical section. Note multiple pyrenoids (p) and strong longitudinal striations in the chloroplast periphery. **(E)**-**(I) ***C. angeleri* Nozaki sp. nov. 2010-0126-1. **(E)**, **(F)** Two views of a 32-celled colony shown at the same magnification. **(E)** Surface view. **(F)** Optical section. **(G)**-**(I)** Three views of cells showing contractile vacuoles (cv) and pyrenoids (p), shown at the same magnification throughout.

Two species of the new colonial volvocine genus *Colemanosphaera* showed essentially the same colonial organization during the vegetative phase (Figure 1). Vegetative colonies were ovoid to ellipsoidal in shape in *C. charkowiensis* comb. nov. (Figure 1A, B) or cylindrical to elongate-ovoid in *C. angeleri* sp. nov. (Figure 1E, F). They contained 16 or 32 cells of approximately identical size embedded at the peripheral regions of the gelatinous matrix, forming a hollow colonial structure. The 32-celled colonies of *C. charkowiensis* measured up to 85 μm long and 67 μm wide, whereas those of *C. angeleri* were up to 95 μm long and 70 μm wide. The cells were ovoid to subspheroidal in *C. charkowiensis* or subspheroidal to lenticular in *C. angeleri*, having a broad anterior face that was more or less angular by mutual compression, measuring up to 20 μm in surface diameter in both species (Figure 1). Each cell had two equal flagella, a massive cup-shaped chloroplast, a stigma, and two or three contractile vacuoles near the base of the flagella (Figure 1C, G, I). No other contractile vacuoles were distributed on the surface of the protoplast. A gradual decrease in stigma size occurred from the anterior to posterior pole of the colony. The chloroplast contained longitudinal striations on the surface (Figure 1C, G), which were prominent in *C. charkowiensis* (Figure 1A-D). Although the chloroplast of both species contained only a single pyrenoid in the immature cells, three to eight pyrenoids of almost identical size were distributed throughout the chloroplast of mature vegetative cells in *C. charkowiensis* (Figure 1D), whereas the cup-shaped chloroplast in mature cells of *C. angeleri* contained a large single pyrenoid in the bottom, as well as one to five small pyrenoids (Figure 1H, I).

**Figure 2:**
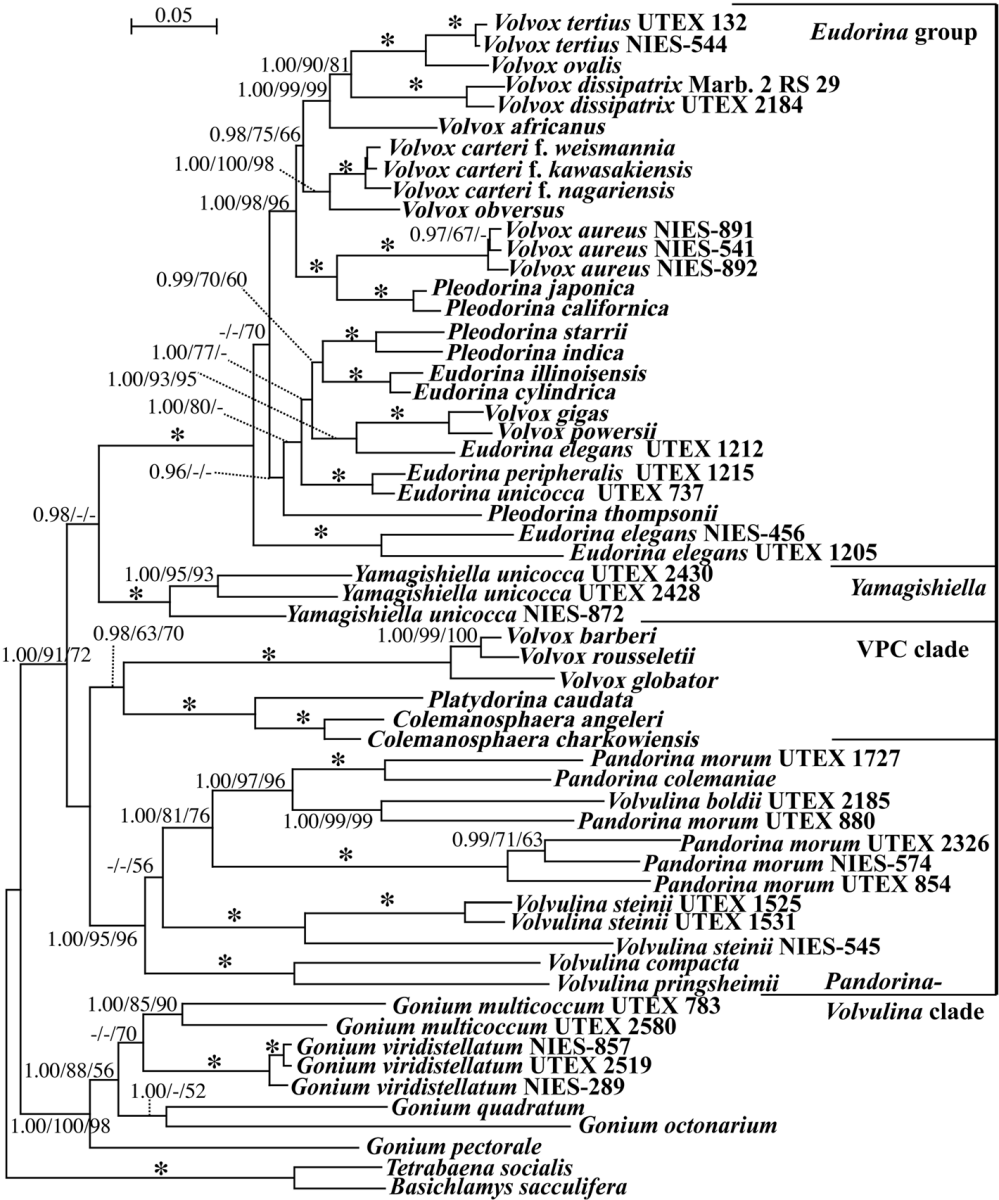
**Bayesian inference (BI) tree of the Volvocaceae based on the chloroplast five genes.** For details of the methods, see the Methods Section. Branch lengths are proportional to the genetic distances, which are indicated by the scale bar above the tree. Numbers on the left, middle, or right side at the branches represent posterior probabilities (PP) of BI (≥0.95), bootstrap values (≥50%, based on 1,000 replicates) obtained with the maximum likelihood and maximum parsimony analyses, respectively. Asterisks at the branches indicate 1.00 PP and 100% bootstrap values by the two methods.

The present two species of *Colemanosphaera* represent vegetative and reproductive morphological attributes that are characteristic of the Volvocaceae [7,12] (Additional file 1: Figures S1 and S2; Additional file 2: Information S1), including 1) cruciate eight-celled plakea (Additional file 1: Figure S1C), 2) typical inversion during daughter colony formation (Additional file 1: Figure S1A), and 3) tripartite colonial boundary of the extracellular matrix in vegetative colonies under transmission electron microscopy (TEM) (Additional file 1: Figure S2). In addition, the present molecular phylogeny confirmed that this new genus is robustly positioned within the Volvocaceae (Figure 2; Additional file 2: Information S1).

**Figure 3:**
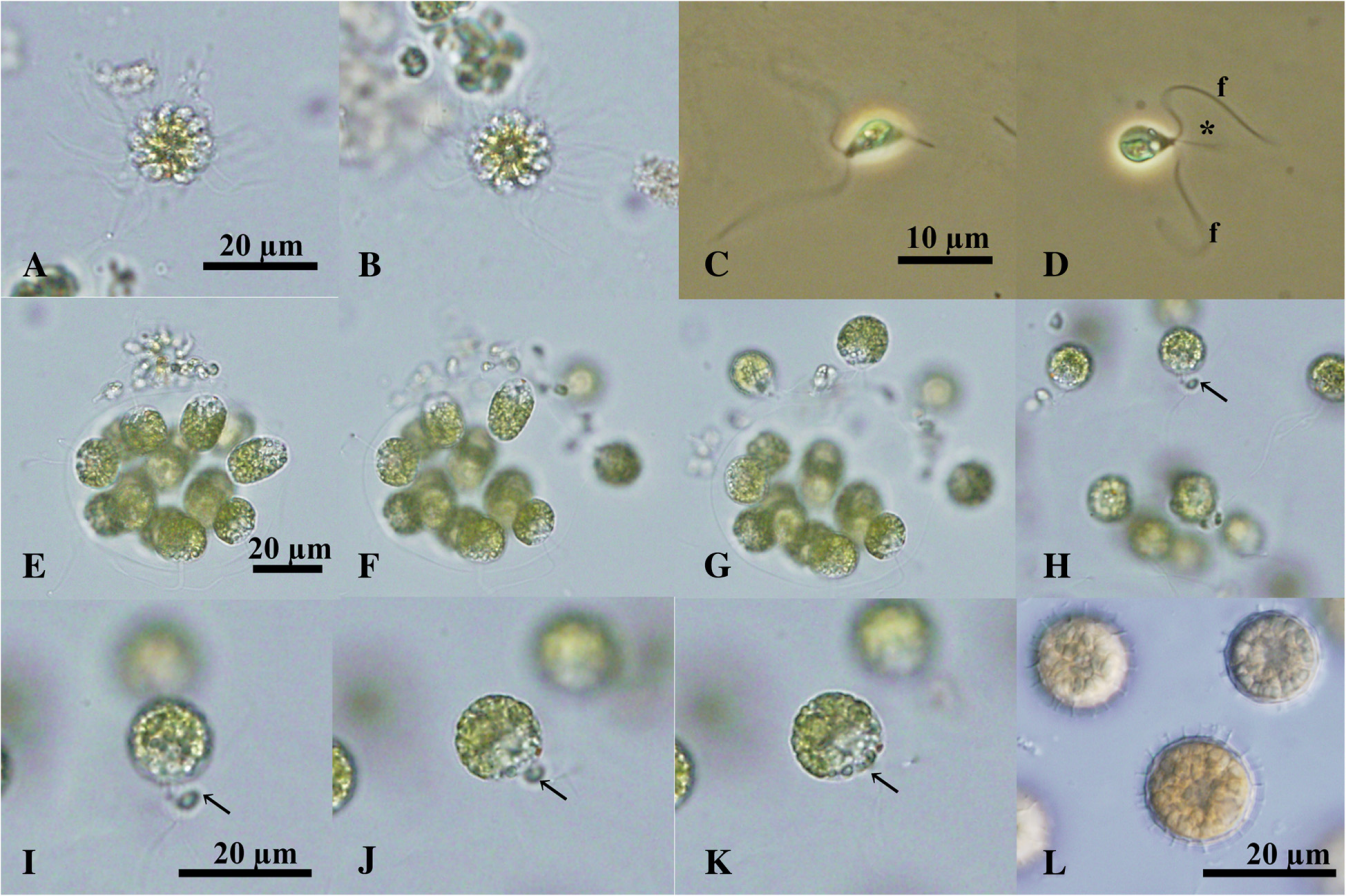
**Light microscopy of sexual reproduction in *Colemanosphaera charkowiensis *(Korshikov) Nozaki et al. comb. nov. (A), (B), (E)-(L)** 2013-0615-IC-3x4x7. **(C)**, **(D)** 2010-0713-E5. **(A)**, **(B)** Sperm packets (bundles of male gametes) shown at the same magnification. **(C)**, **(D)** Male gametes shown at the same magnification. Note cytoplasmic protrusion (asterisk) near the base of the flagella **(f). (E)-(K)** Successive stages of male and female gamete release and conjugation. Note male gamete (arrow) fusing with female gamete. **(E)-(H)** Shown at the same magnification throughout. **(I)-(K)** Shown at the same magnification throughout. **(L)** Mature aplanozygotes.

Within the Volvocaceae, *Colemanosphaera* is morphologically similar to *Yamagishiella* and *Eudorina* in having 16- or 32-celled spheroidal vegetative colonies without differentiation of obligative somatic cells, but with a cellular envelope that encompasses each vegetative cell tightly inside the colonial boundary under TEM [12,13] (Additional file 1: Figure S2). However, *Colemanosphaera* and *Yamagishiella* can be clearly distinguished from *Eudorina* based on differences in contractile vacuoles in vegetative cells. Small contractile vacuoles are distributed on the surface of the vegetative cells of *Eudorina*, whereas vegetative cells of *Yamagishiella* and *Colemanosphaera* contain only two or three contractile vacuoles near the base of the flagella [14] (Figure 1). Furthermore, *Colemanosphaera* differed from *Yamagishiella* in pyrenoid characteristics and sexual reproduction. *Colemanosphaera* contained multiple pyrenoids in the chloroplast of mature vegetative cells and showed anisogamous sexual reproduction with sperm packets (Figures 1 and 3). *Yamagishiella unicocca,* the only species of the genus, was described as having a single basal pyrenoid in the chloroplast and isogamous sexual reproduction [12,14,15]. Therefore, *Colemanosphaera* is a previously undescribed morphological genus within the Volvocaceae.

**Figure 4:**
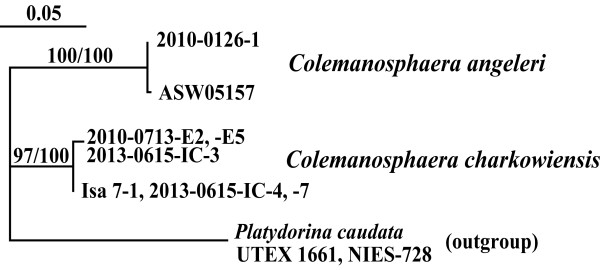
**Maximum likelihood (ML) tree of two species of *Colemanosphaera *and an Austrian strain (ASW05157) based on internal transcriber spacer region of nuclear ribosomal DNA.** For details of the methods, see the Methods Section. Branch lengths are proportional to the genetic distances, which are indicated by the scale bar above the tree. Numbers on the left or right side at the branches represent bootstrap values (≥50%, based on 1,000 replicates) obtained with the ML and maximum parsimonious analyses, respectively.

The present two species of *Colemanosphaera* can be clearly distinguished based on differences in chloroplast morphology, development of flagellar elongation in newly formed daughter colonies, and molecular data. The chloroplast in *C. charkowiensis* shows prominent longitudinal striations on the surface and has more than two pyrenoids of almost identical size in mature cells, whereas *C. angeleri* has chloroplast striations that are not as prominent and contains a large basal pyrenoid and small pyrenoids in the cup-shaped chloroplast in mature cells (Figure 1). During asexual reproduction, two new flagella in each cell of a newly formed daughter colony are markedly different in length in *C. charkowiensis*, whereas the two flagella of *C. angeleri* grow to an equal length (Additional file 1: Figure S1D, E). In addition, *C. charkowiensis* and *C. angeleri* were robustly separated from each other in the phylogenetic analysis of the ITS region of *r*DNA (Figure 4), and two compensatory base changes (CBCs) were detected in helix I and helix III of ITS-2 of the *r*DNA between these two species (Additional file 1: Figures S3-S6; Additional file 2: Information S1). The latter CBC is positioned within the most conserved region of ITS-2 of *r*DNA (the contiguous 5′-side 30 nucleotide positions of the distal portion of helix III) [16]. According to Coleman [16], organisms that differ by even one CBC in this region also are completely unable to cross. Thus, the two species represent two genetically and morphologically distinct entities.

The Austrian strain ASW05157 and *C. angeleri* 2010-0126-1 formed a small clade robustly separated from *C. charkowiensis* in the phylogenetic analysis of the ITS region of *r*DNA (Figure 4), and showed only one nucleotide difference in the ITS region (Additional file 2: Information S1). The ITS region of nuclear *r*DNA is rapidly evolving and shows nucleotide variation within a single biological species [16]. Thus, ASW05157 unambiguously belongs to *C. angeleri*, although its morphological data are lacking.

### Sexual reproduction

Sexual reproduction was observed in *C. charkowiensis* (Figure 3) and generally occurred 2 days after possible male and female cultures were mixed in the nitrogen-deficient mating medium [17]. Cells of the colony divided to form a bundle of 16 or 32 male gametes or sperm packets. The packet was almost spherical in shape, measured 15–17 μm in diameter (Figure 3A, B), and swam to the female colony, which was almost indistinguishable from the vegetative colony. The packet on the female colony eventually dissociated into individual, biflagellate male gametes. The male gametes were spindle-shaped to elongate-ellipsoidal in shape and measured 5–10 μm in length. A tubular cytoplasmic protrusion was observed near the base of the flagella of the male gametes (Figure 3C, D), similar to male gametes of *Eudorina elegans*[18]. During dissociation of the packet, biflagellate protoplasts or female gametes escaped from the gelatinous matrix of the female colony (Figure 3E-G). Female gametes were biflagellate and spherical in shape, measuring 13–17 μm in diameter. Female and male gametes fused outside the female colony (Figure 3H–K). The anterior end of the male gamete connected the anterior region of the female gamete, and the plasmogamy proceeded to form a quadriflagellate zygote. The zygotes then lost their flagella and secreted a reticulate cell wall (Figure 3L). The zygotes eventually turned reddish brown in color after about 1 week. The mature aplanozygotes were 14–18 μm in diameter (excluding reticulation).

**Figure 5:**
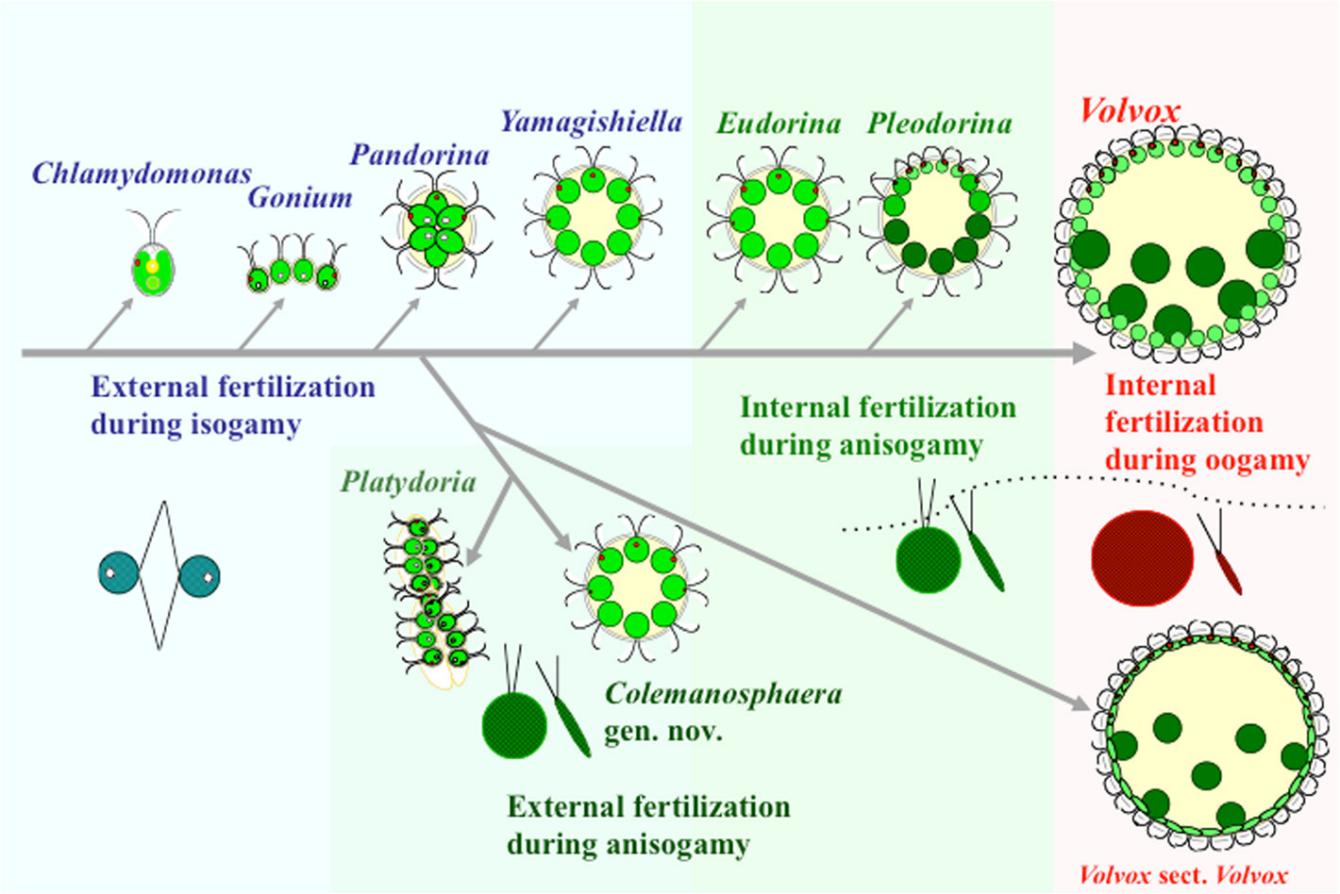
**Diagram showing phylogeny and evolution of sexual reproduction characteristics within the colonial volvocine greens.** Based on the present study.

In *C. charkowiensis*, male colonial cells underwent successive cell divisions to form bundles of spindle-shaped male gametes (sperm packets), whereas female gametes formed without successive cell divisions of colonial cells (Figure 3). These characteristics are essentially the same as in other members of the anisogamous/oogamous volvocacean genera *Eudorina*, *Pleodorina*, *Platydorina*, and *Volvox*[7]. However, *C. charkowiensis* differed from dioecious members of *Eudorina*, *Pleodorina*, and *Volvox* during conjugation between male and female gametes. Male and female gametic union in the latter genera takes place within the female colony after penetration by the male gametes [13,17-21]. In contrast, female gametes are released from the colonial matrix, and male and female gametes fuse outside the female colony in *C. charkowiensis* (Figure 3). A similar “external fertilization” between male and female motile gametes was observed in natural-collected samples of *Platydorina*[22]. This type of anisogamous conjugation is similar to isogamous conjugation in *Pandorina*, *Volvulina*, and *Yamagishiella* in that the gametes of both sexes are released from the parental colonies [21]. In addition, the present multigene phylogeny demonstrated that *Colemanosphaera* and *Platydorina* form a robust clade that is sister to the lineage of *Volvox* sect. *Volvox* (Figures 2 and 5), which shows “internal fertilization during oogamy” [7,23]. Thus, “external fertilization during anisogamy,” as found in *Colemanosphaera* and *Platydorina*, may represent an evolutionary intermediate between “external fertilization during isogamy” and “internal fertilization during anisogamy/oogamy” in the colonial volvocine green algae (Figure 5).

### Phylogenetic significance

The present multigene phylogeny demonstrated that *Colemanosphaera* is robustly sister to the unique flattened volvocacean genus *Platydorina* (Figure 2). Although these genera differ in the form of their vegetative colonies (spheroidal vs. flattened, respectively), they share fundamental morphological characteristics during the vegetative phase and sexual reproduction. The vegetative colonies of both genera are composed of 16 or 32 cells of identical size [7,22,24] (Figure 1). Two or three contractile vacuoles are limited to the anterior end of the vegetative cells in both genera (Figure 1; Additional file 1: Figure S7). Vegetative ultrastructures of these two genera were used to observe colonial boundaries and cellular envelopes [12] (Additional file 1: Figure S2). In addition, both genera show sperm packet formation and “external fertilization” between male and female motile gametes [22] (Figure 3). Thus, *Colemanosphaera* may exhibit ancestral morphological attributes of *Platydorina*, and a *Colemanosphaera*-like spheroidal colonial ancestor might have evolved to *Platydorina* by acquiring intercalation to form flattened vegetative colonies during embryogenesis.

### Taxonomic consideration of species

Although small contractile vacuoles are distributed on the surface of the vegetative cells of *Eudorina*[14], Huber-Pestalozzi [25] and Ettl [26] characterized the genus *Eudorina* as having two anterior contractile vacuoles. Thus, some *Eudorina* species in which contractile vacuoles have not been studied carefully may actually belong to *Colemanosphaera.* Among the described species of *Eudorina*[19,27], *E. echidna* and *E. interconnexa* have not been studied in culture. In addition, the strain of *E. conradii*[28] is not available from the UTEX Culture Collection (http://web.biosci.utexas.edu/utex/). However, these three species of *Eudorina* can be clearly distinguished from the present two species of *Colemanosphaera* based on differences in vegetative morphology. These three *Eudorina* species have almost spherical vegetative cells [19,29,30]. In contrast, cells of two species of *Colemanosphaera* are ovoid, subspheroidal, or lenticular in shape with a broad anterior face that seems more or less angular by mutual compression (Figure 1). In addition, the three species of *Eudorina* are characterized by distinctive vegetative colony structures [19]. *E. conradii* and *E. interconnexa* have species-specific individual gelatinous sheaths surrounding vegetative cells [19,30], while *E. echidna* shows short conical projections covering the vegetative colony [29].

Thompson [31] observed conjugation between small male and large female gametes outside the female colony in “*Pandorina charkowiensis.*” However, male gametes are not elongate or spindle-shaped in this alga. Although *P. charkowiensis* contains multiple pyrenoids in the cup-shaped chloroplast in each vegetative cell [32] (Figure 1), information on pyrenoids of the alga observed by Thompson [31] is lacking in his description and figures. Therefore, the alga may be *Yamagishiella unicocca*, in which small and large gametes can fuse [15,18].

Korshikov’s description of the alga from the Ukraine that he called *Pandorina charkowiensis* indicates that it is highly similar in vegetative morphology to the Japanese *C. charkowiensis* strains described here, in that it has subspheroidal cells with a broad anterior face, two contractile vacuoles, and multiple (up to seven) pyrenoids in a cup-shaped chloroplast with prominent longitudinal striations on its surface [32]. He did not evaluate its sexual reproduction [32], but if he had, there is little doubt that he would have found that it was not isogamous like *Pandorina*. On the other hand, the other *Colemanosphaera* species (*C. angeleri*) contains more flattened vegetative cells and less prominent chloroplast striations than those of *C. charkowiensis* (Figure 1). Thus, *C. angeleri* represents a previously undescribed morphological species.

## Conclusions

Two species of *Colemanosphaera* were found in a Japanese lake (Additional file 2: Table S1). As discussed above, comparison of ITS sequences demonstrated that the Austrian strain ASW05157 unambiguously belongs to *C. angeleri* (Figure 4). *C. charkowiensis* was originally described based on material originating from Ukraine [32]. Thus, *Colemanosphaera* may be distributed in worldwide freshwater habitats. Although *P. charkowiensis* was reported from the United States [31,33,34], it may be *Y. unicocca* or *Eudorina* species because information on contractile vacuoles and/or pyrenoids is limited. Further detailed molecular and morphological studies of the *Eudorina*- and *Yamagishiella*-like colonial volvocine algae will resolve actual worldwide distribution and diversity of the new genus *Colemanosphaera*.

The present study unambiguously demonstrated external fertilization between male and female gametes using *Colemanosphaera* cultures. When such a type of gametic union is considered with previously published data [7], sexual reproduction of the volvocine green algae can be clearly distinguished into four types (with the exception of monoecism) (Figure 5): 1) External fertilization during isogamy with gametes directly released from colonial cells, 2) external fertilization during anisogamy with the formation of bundles of spindle-shaped male gametes (sperm packet formation), 3) internal fertilization during anisogamy (with flagellated female gametes retained within the parental female colony), and 4) internal fertilization during oogamy (with flagella-lacking female gametes formed within the parental female colony). Thus, oogamy evolution may have been preceded by the transition from external to internal fertilization during anisogamy within the volvocine green algae.

### Taxonomic treatments

*Colemanosphaera* Nozaki gen. nov.

Vegetative colonies spheroidal in shape, composed of 16 or 32 cells of approximately identical sizes embedded at the peripheral regions of the gelatinous matrix forming a hollow colonial structure. The extracellular matrix of vegetative colonies exhibiting colonial boundaries and cellular envelopes. Each cell biflagellate, more or less angular in front view, containing a massive cup-shaped chloroplast, a stigma, and a nucleus. Two or three contractile vacuoles distributed near the base of the flagella. Chloroplasts containing longitudinal striations on the surface and multiple pyrenoids in mature cells. A gradual decrease in stigma size occurring from the anterior to posterior pole of the colony. Asexual reproduction accomplished by successive divisions of each vegetative cell, forming a square plakea undergoing typical inversion to produce a spheroidal daughter colony.

Type species: *Colemanosphaera charkowiensis* (Korshikov) Nozaki, T. K. Yamada, F. Takahashi, Matsuzaki et Nakada comb. nov.

Etymology: The generic name “*Colemanosphaera*” honoring Dr. Annette W. Coleman who contributed much to the species systematics of microalgae [16].

*Colemanosphaera charkowiensis* (Korshikov) Nozaki, T. K. Yamada, F. Takahashi, Matsuzaki et Nakada comb. nov.

Basionym: *Pandorina charkowiensis* Korshikov 1923 [32]: 174, pl. 7, Figure 1.

Synonym: *Eudorina charkowiensis* (Korshikov) Pascher 1927 [35]: 441.

Holotype: Figure 1 of pl. 7 in Korshikov [32].

Epitype here designated: Resin-embedded vegetative spheroids of 2013-0615-IC-7 (= NIES-3388) deposited in the herbarium of the Department of Botany, National Museum of Nature and Science (TNS), Tsukuba, Japan (TNS-AL-56994).

Strains examined: 2013-0615-IC-7, 2013-0615-IC-4, 2013-0615-IC-3, Isa 7–1, 2010-0713-E2, and 2010-0713-E5 (Additional file 2: Table S1).

Distribution: Ukraine [32], Japan.

*Colemanosphaera angeleri* Nozaki sp. nov.

Vegetative colonies 16- or 32-celled, cylindrical to elongate-ovoid in shape, measuring up to 95 μm long and up to 70 μm wide. Cells subspheroidal to lenticular in shape, measuring up to 20 μm in surface diameter. Chloroplasts in mature cells containing a large pyrenoid and one to five small pyrenoids. Two flagella in each cell of a newly formed daughter colony growing equally. Sexual reproduction not observed.

Holotype: Resin-embedded vegetative spheroids of 2010-0126-1 (= NIES-3382) deposited in the herbarium of the Department of Botany, National Museum of Nature and Science (TNS), Tsukuba, Japan (TNS-AL-56995).

Strain examined: 2010-0126-1 (Additional file 2: Table S1).

Etymology: The species epithet “*angeleri*” honoring Dr. David Angeler who studied the Austrian strain ASW05157 of this species [11].

Type locality: Lake Isanuma, Isanuma, Kawagoe-shi, Saitama, Japan (Additional file 2: Table S1).

Distribution: Austria [11], Japan.

## Methods

Samples of *Colemanosphaera* were collected four times during the last several years from Lake Isanuma and its adjoining small pond, Isanuma, Kawagoe-shi, Saitama Prefecture, Japan (Additional file 2: Table S1). Clonal cultures were established using the pipette-washing method [36] either directly from water samples or from media obtained by rewetting a small amount of dried soil in a 90- × 20-mm Petri dish. The cultures were grown in screw-cap tubes (18 × 150 mm) containing ~11 mL of AF-6 medium [37] modified by Kasai *et al*. [38] or VTAC medium (containing 200 mg L^−1^ sodium acetate 4H_2_O [18,38]) at 23-25°C on a 14-h light:10-h dark schedule under cool-white fluorescent lamps (color temperature = 4000–5000 K) at an intensity of 110–150 μmol∙m^−2^∙s^−1^. Vegetative colonies and asexual reproduction were observed by examining a small aliquot of cells grown continuously by inoculating ~0.5–1.0 mL of actively growing cells into fresh medium every 3–10 days.

To induce sexual reproduction, cultures grown in the modified AF-6 medium at about 25°C were concentrated from 11 to 0.5 mL by centrifugation. The concentrated possible male and female cultures were mixed with 10 mL of nitrogen-deficient “mating medium” [17] in a 60 × 10-mm Petri dish.

To examine the light microscopic details and the ITS region of nuclear *r*DNA, *Platydorina caudata* UTEX 1661 and NIES-728 (= UTEX 1658) were obtained from the Culture Collection of Algae at the University of Texas at Austin [39] (http://web.biosci.utexas.edu/utex/) and Microbial Culture Collection at the Institute for National Environmental Studies [38] (http://mcc.nies.go.jp/), respectively, and they were cultured in the modified AF-6 medium as described above. Light microscopy was performed using a BX60 microscope (Olympus, Tokyo, Japan) equipped with Nomarski optics. TEM was performed using 0.8% glutaraldehyde during pre-fixation as described previously [12].

Five chloroplast genes (*atpB*, *rbcL*, *psaA*, *psaB*, and *psbC*) and ITS region of *r*DNA (ITS-1, 5.8S *r*DNA, and ITS-2) of two species of *Colemanosphaera* and the ITS region of *P. caudata* UTEX 1661 and NIES-728 were sequenced as described previously [3,40]. For phylogenetic analysis of chloroplast genes, the combined coding regions of the five chloroplast genes [3] from 58 operational taxonomic units (OTUs) of the colonial volvocine algae (Additional file 2: Table S2) were subjected to Bayesian inference using MrBayes 3.2.1. [41] as described previously [42]. In addition, 1,000 replicates of bootstrap analyses [43] were performed by maximum-likelihood (ML) analysis with RAxML ver. 7.0.4 [44] using a GTR model of each codon position in the five concatenated genes unlinked and by the maximum-parsimony (MP) method using PAUP 4.0b10 [45], as described previously [46]. Because the third codon positions of the combined five genes showed heterogeneous base compositions among OTUs (p < 0.01) based on chi-square tests of PAUP 4.0, the third codon positions of *psaB* genes, which seemed to have the greatest effect on the heterogeneity among the five genes, were excluded to minimize heterogeneity (p > 0.05). The alignment (5,525 positions and 38 OTUs) used for the present phylogenetic analyses (Figure 2) is available from TreeBASE (http://treebase.org/treebase-web/home.html; study ID: S15331).

Goniacean and tetrabaenacean OTUs were designated as the outgroup because Volvocaceae is monophyletic and sister to the Goniaceae, and these two families and the Tetrabaenaceae form a large monophyletic group [3-5]. Because the colonial volvocine *Astrephomene* species (Goniaceae) showed possible saturation of the chloroplast genes [3] and were positioned outside the Volvocaceae [3,5], we excluded this genus.

Nucleotide sequences for the ITS region of *r*DNA from *Colemanosphaera* (Additional file 2: Table S1), ASW05157, and the outgroup *P. caudata* UTEX 1661 and NIES-728 (accession numbers AB905587 and AB905588) were aligned using ClustalX [47]. After refining the alignment based on the secondary structures of ITS-1 and ITS-2 [10,48] and reducing identical sequences to a single OTU, the alignment (676 positions and five OTUs; study ID: S15331 available from TreeBASE) were subjected to MP and ML (based on HKY85 model) methods based on a branch-and-bound search by PAUP 4.0 with bootstrap analysis based on 1,000 replicates. The secondary structures of ITS-2 were predicted based on that of *Platydorina*[48] and refined as described previously [49] using Centroidfold [50] and RNAfold at the RNAfold WebServer [51] (http://rna.tbi.univie.ac.at/cgi-bin/RNAfold.cgi).

## Abbreviations

BI: Bayesian inference; CBC: Compensatory base changes; ITS: Internal transcriber spacer; ML: Maximum likelihood; MP: Maximum parsimony; OTU: Operational taxonomic unit; *r*DNA: Ribosomal DNA; TEM: Transmission electron microscopy.

## Competing interests

The authors declare that they have no competing interests.

## Authors’ contributions

HN, TKY and FT conducted the morphological and molecular biology experiments. HN, RM and TN constructed phylogenetic trees and secondary structures of nuclear ITS2. HN and RM wrote the manuscript. All authors read and approved the manuscript.

## Supplementary Material

Additional file 1: Figure S1 Light microscopy of asexual reproduction of two species of *Colemanosphaera.***Figure S2.** Transmission electron micrographs of vegetative colony of two species of *Colemanosphaera*. **Figure S3.** Secondary structures of helix I and helix III of nuclear ribosomal DNA internal transcribed spacer 2 transcript of *Colemanosphaera charkowiensis* (Korshikov) Nozaki et al. comb. nov. (Isa 7–1, 2010-0713-E2, 2010-0713-E5, 2013-0615-IC-3, 2013-0615-IC-4 and 2013-0615-7), and *C. angeleri* Nozaki sp. nov. (2010-0126-1 and ASW05157). **Figure S4.** Whole secondary structure of nuclear ribosomal DNA internal transcribed spacer 2 transcript of *Colemanosphaera charkowiensis* (Korshikov) Nozaki et al. comb. nov (2010-0713-E2, 2010-0713-E5 and 2013-0615-IC-3). **Figure S5.** Whole secondary structure of nuclear ribosomal DNA internal transcribed spacer 2 transcript of *Colemanosphaera angeleri* Nozaki sp. nov. (2010-0126-1 and ASW05157). **Figure S6.** Whole secondary structure of nuclear ribosomal DNA internal transcribed spacer 2 transcript of *Platydorina caudata* Kofoid UTEX 1661 and NIES-728 (=UTEX 1658). **Figure S7.** Light microscopy of vegetative colonies of *Platydorina caudata* Kofoid. NIES-728 (=UTEX 1658). Click here for file

Additional file 2 **Information S1.** Asexual reproduction, transmission electron microscopy, molecular phylogenetic analyses, and secondary structures of ITS-2 *r*DNA. **Table S1.** List of strains of *Colemanosphaera* used in this study. **Table S2.** List of the colonial volvocine taxa/strains included in the phylogenetic analysis (Figure 2) and DDBJ/EMBL/GenBank accession numbers of the five chloroplast genes. Click here for file

## Data depositions

All new sequences have been deposited to DDBJ/EMBL/GenBank (accession numbers: AB905580 - AB905598). Sequence alignments of the five chloroplast genes and the ITS region of nuclear *r*DNA have been deposited in TreeBASE (study ID: S15331).
