# Supplementary material for: New “missing link” genus of the colonial volvocine green algae gives insights into the evolution of oogamy
Source: BMC Evol Biol. 2014 Mar 3;14:37. doi: 10.1186/1471-2148-14-37 (PMC4015742; doi:10.1186/1471-2148-14-37)
Supplement: Additional file 1: Figure S1 — Light microscopy of asexual reproduction of two species of Colemanosphaera.Figure S2. Transmission electron micrographs of vegetative colony of two species of Colemanosphaera. Figure S3. Secondary structures of helix I and helix III of nuclear ribosomal DNA internal transcribed spacer 2 transcript of Colemanosphaera charkowiensis (Korshikov) Nozaki et al. comb. nov. (Isa 7–1, 2010-0713-E2, 2010-0713-E5, 2013-0615-IC-3, 2013-0615-IC-4 and 2013-0615-7), and C. angeleri Nozaki sp. nov. (2010-0126-1 and ASW05157). Figure S4. Whole secondary structure of nuclear ribosomal DNA internal transcribed spacer 2 transcript of Colemanosphaera charkowiensis (Korshikov) Nozaki et al. comb. nov (2010-0713-E2, 2010-0713-E5 and 2013-0615-IC-3). Figure S5. Whole secondary structure of nuclear ribosomal DNA internal transcribed spacer 2 transcript of Colemanosphaera angeleri Nozaki sp. nov. (2010-0126-1 and ASW05157). Figure S6. Whole secondary structure of nuclear ribosomal DNA internal transcribed spacer 2 transcript of Platydorina caudata Kofoid UTEX 1661 and NIES-728 (=UTEX 1658). Figure S7. Light microscopy of vegetative colonies of Platydorina caudata Kofoid. NIES-728 (=UTEX 1658). [file 1471-2148-14-37-S1.pdf]

Additional file 1:

Figures S1-S7

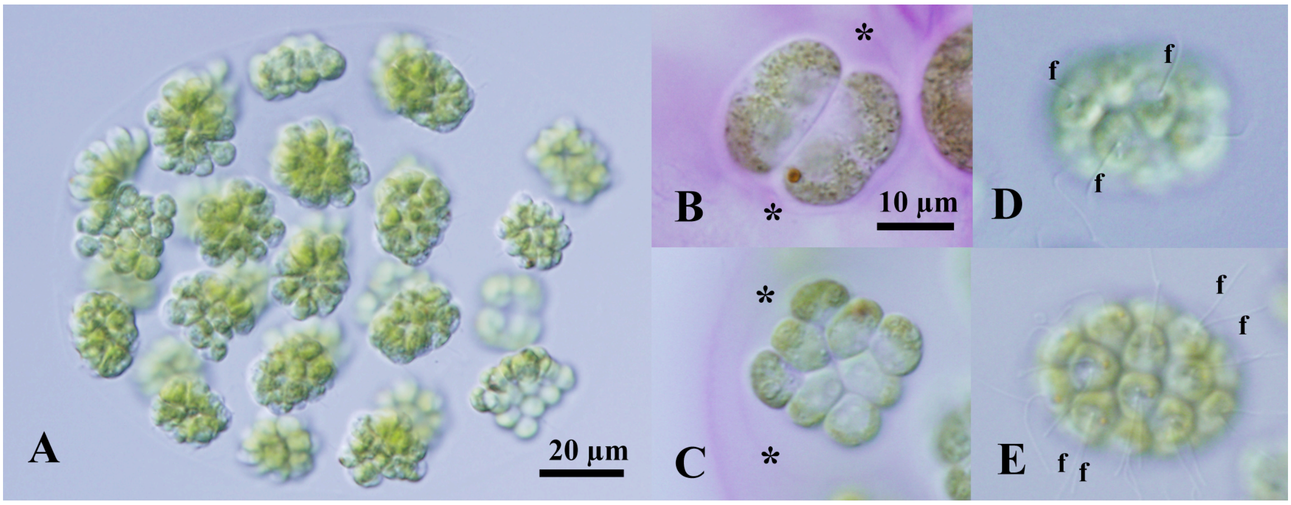

Figure S1. Light microscopy of asexual reproduction of two species of *Colemanosphaera*. (A) Inversion stages and newly formed daughter colonies in *C. charkowiensis* (Korshikov) Nozaki et al. comb. nov. 2010-0713-E2. (B) Two-celled embryo enclosed by transparent vesicle (asterisks) in *C. angeleri* Nozaki sp. nov. 2010-0126-1. (C) Eight-celled embryo enclosed by transparent vesicle (asterisks) in *C. charkowiensis* (Korshikov) Nozaki et al. comb. nov. 2010-0713-E2. (D) Newly formed daughter colony in *C. charkowiensis* (Korshikov) Nozaki et al. comb. nov. 2010-0713-E5. Note only one long flagellum (f) in each protoplast. (E) Newly formed daughter colony in *C. angeleri* Nozaki sp. nov. 2010-0126-1. Note two long flagella (f) in each protoplast. Scale bar in (B) applies to (C)-(E).

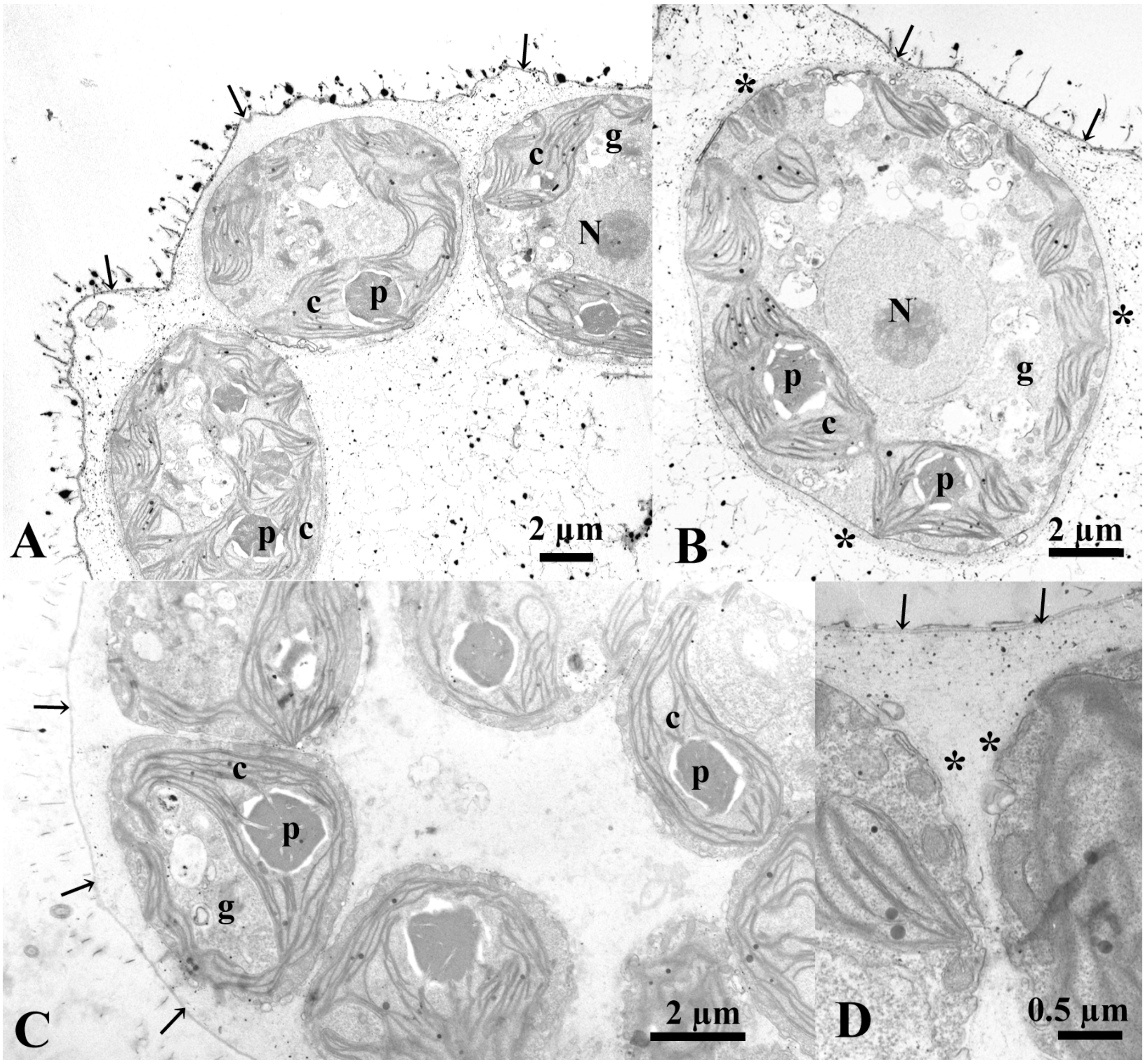

Figure S2. Transmission electron micrographs of vegetative colony of two species of *Colemanosphaera*. (A), (B) *C. charkowiensis* (Korshikov) Nozaki et al. comb. nov. Isa 7-1. (C), (D) *C. angeleri* Nozaki sp. nov. 2010-0126-1. Note that each cell is enclosed by a cellular envelope (asterisks) within the colonial boundary (arrows) of the extracellular matrix. c, chloroplast; g, Golgi body; N, nuclus; p, pyrenoid.

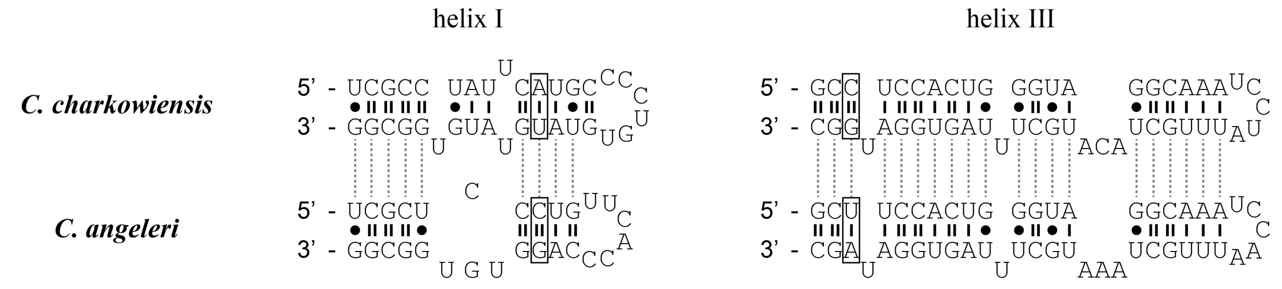

Figure S3. Secondary structures of helix I and helix III of nuclear ribosomal DNA internal transcribed spacer 2 transcript of *Colemanosphaera charkowiensis* (Korshikov) Nozaki et al. comb. nov. (Isa 7-1, 2010-0713-E2, 2010-0713-E5, 2013-0615-IC-3, 2013-0615-IC-4 and 2013-0615-7), and *C. angeleri* Nozaki sp. nov. (2010-0126-1 and ASW05157). Base pairs enclosed by box represent compensatory base change between the two species.

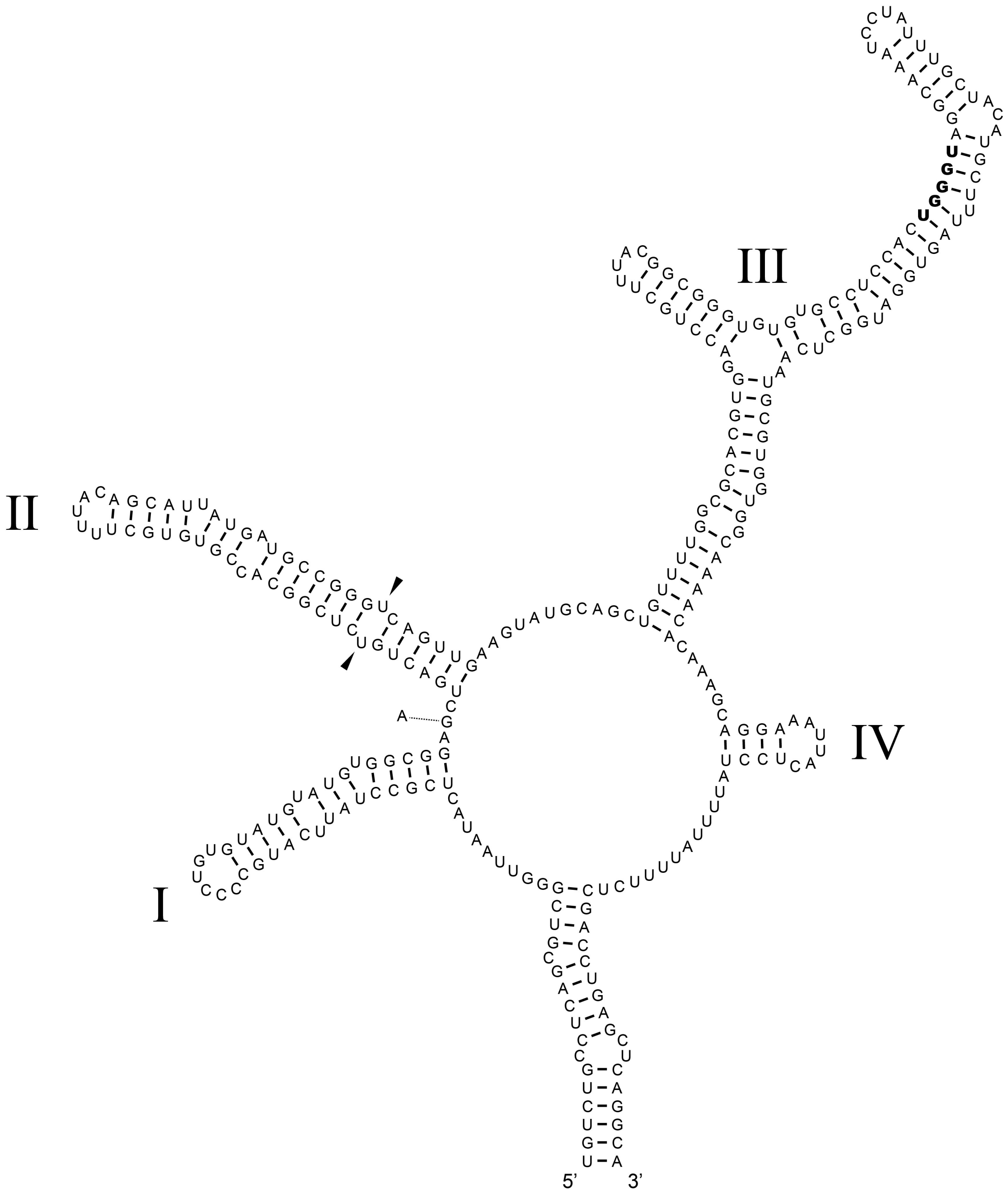

Figure S4. Whole secondary structure of nuclear ribosomal DNA internal transcribed spacer 2 transcript of *Colemanosphaera charkowiensis* (Korshikov) Nozaki et al. comb. nov (2010-0713-E2, 2010-0713-E5 and 2013-0615-IC-3). Note the U-U mismatch in helix II (arrowheads) and the UGGU motif on the 5’ side near the apex of helix III (boldface). The structure was predicted as described in the methods section in the main text and was drawn using Varna version 3.9 [1]. One nucleotide (A in the left side) is different from this sequence in three other strains (Isa 7-1, 2013-0615-IC-4 and 2013-0615-IC-7) of the same species.

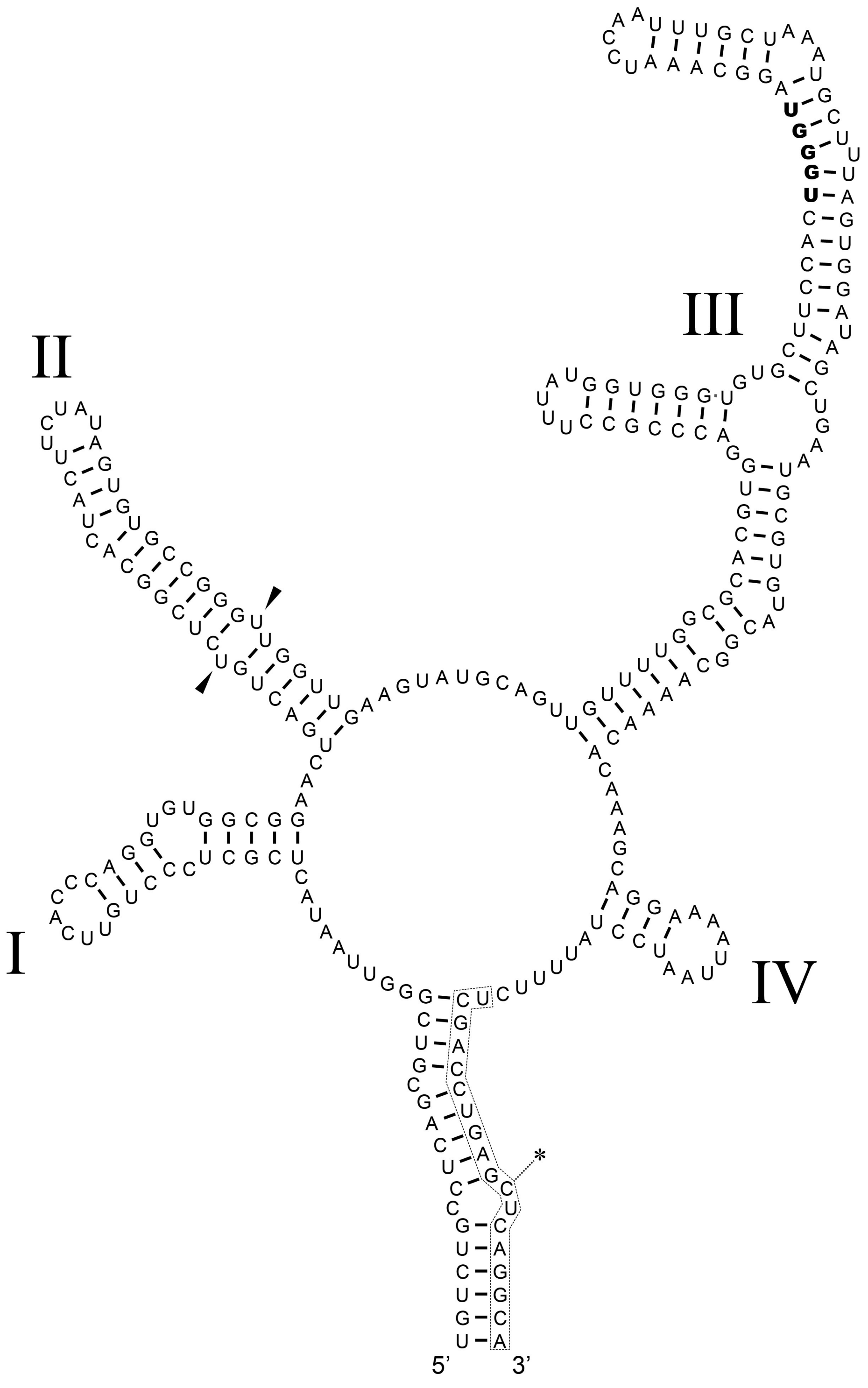

Figure S5. Whole secondary structure of nuclear ribosomal DNA internal transcribed spacer 2 transcript of *Colemanosphaera angeleri* Nozaki sp. nov. (2010-0126-1 and ASW05157). Note the U-U mismatch in helix II (arrowheads) and the UGGU motif on the 5' side near the apex of helix III (boldface). The structure was predicted as described in the methods section in the main text and was drawn using Varna version 3.9 [1]. 3' terminal sequence (asterisk) is missing in ASW05157.

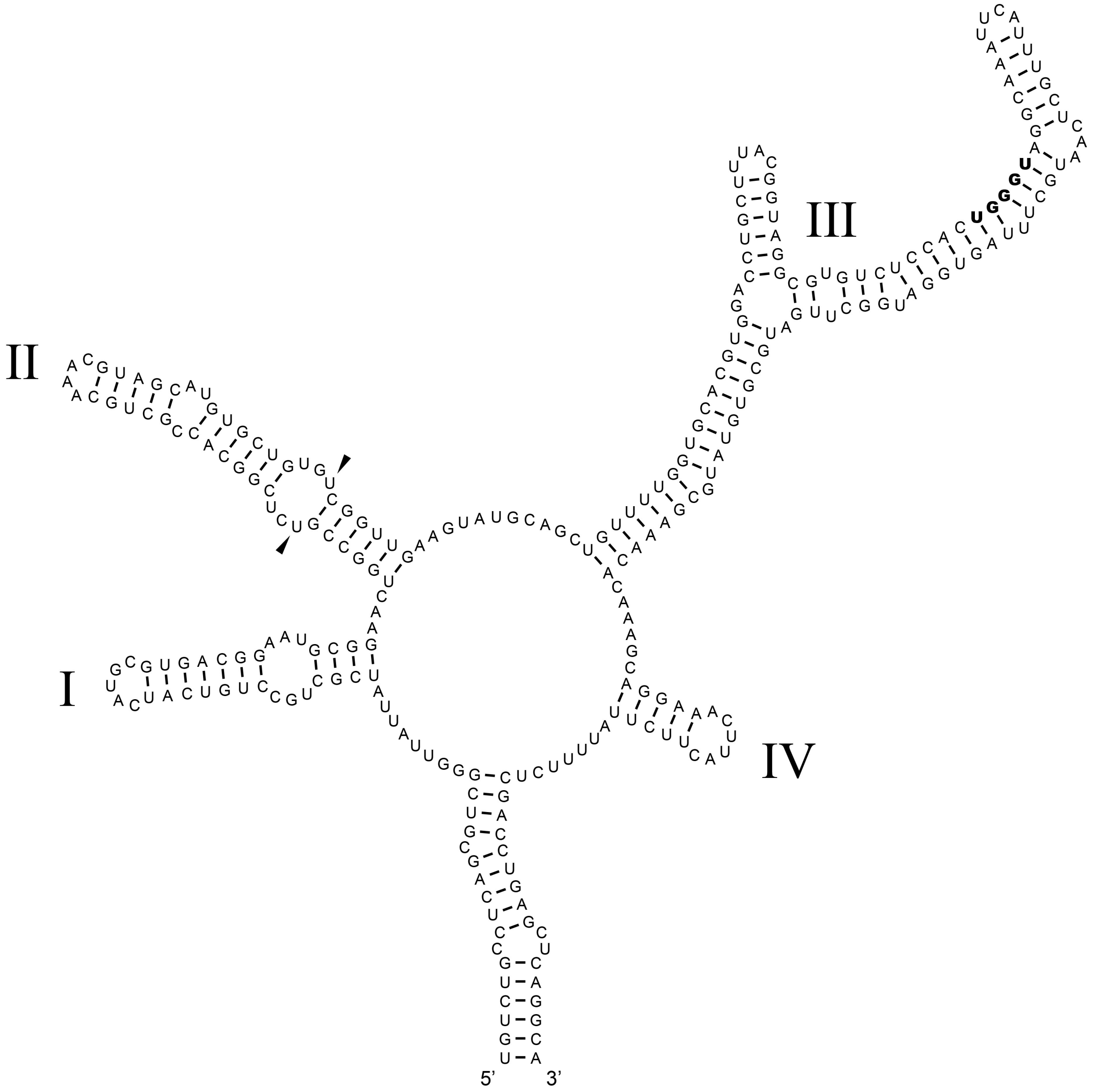

Figure S6. Whole secondary structure of nuclear ribosomal DNA internal transcribed spacer 2 transcript of *Platydorina caudata* Kofoid UTEX 1661 and NIES-728 (=UTEX 1658). Note the U-U mismatch in helix II (arrowheads) and the UGGU motif on the 5’ side near the apex of helix III (boldface). The structure was predicted as described in in the methods section in the main text and was drawn using Varna version 3.9 [1].

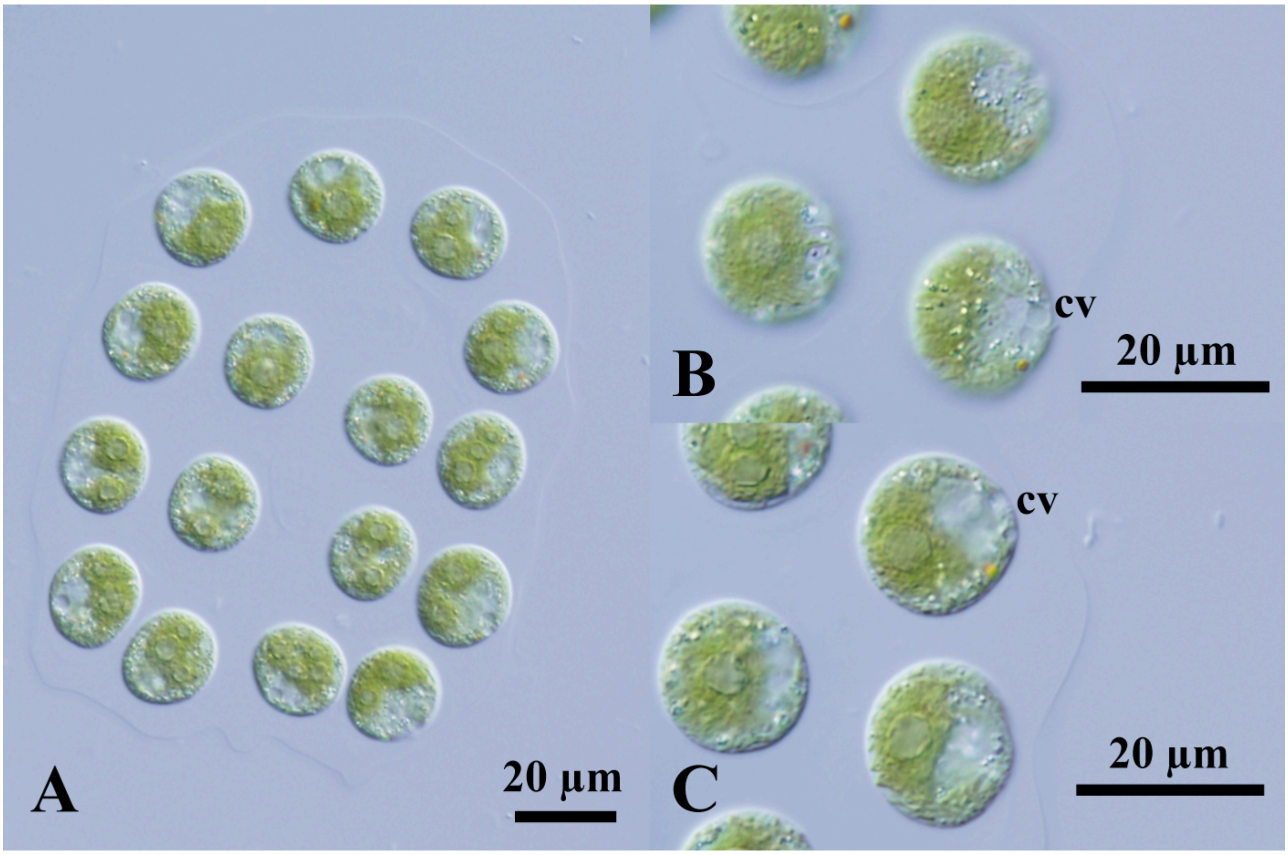

Figure S7. Light microscopy of vegetative colonies of *Platydorina caudata* Kofoid. NIES-728 (= UTEX 1658). Note contractile vacuoles (cv) positioned in only the anterior end of cells.

Reference

1. Darty K, Denise A, Ponty Y: **VARNA: Interactive drawing and editing of the RNA secondary structure.** *Bioinformatics* 2009, **25**: 1974-1975.
