# Supplementary material for: New “missing link” genus of the colonial volvocine green algae gives insights into the evolution of oogamy
Source: BMC Evol Biol. 2014 Mar 3;14:37. doi: 10.1186/1471-2148-14-37 (PMC4015742; doi:10.1186/1471-2148-14-37)
Supplement: Additional file 2 — Information S1. Asexual reproduction, transmission electron microscopy, molecular phylogenetic analyses, and secondary structures of ITS-2 rDNA. Table S1. List of strains of Colemanosphaera used in this study. Table S2. List of the colonial volvocine taxa/strains included in the phylogenetic analysis (Figure 2) and DDBJ/EMBL/GenBank accession numbers of the five chloroplast genes. [file 1471-2148-14-37-S2.pdf]

Additional file 2:

Information S1 and Tables S1 and S2

Information S1. Asexual reproduction, transmission electron microscopy, molecular phylogenetic analyses, and secondary structures of ITS-2 *r*DNA.

Asexual reproduction

During asexual reproduction of the two new species of *Colemanosphaera* (Additional file: Figure S1), each reproductive cell divided four or five times successively to form a 16- or 32-celled square plakea, respectively. In 8-celled plakea, the protoplasts arranged cruciately, similar to other members of the Volvocaceae [1-3]. The plakea then inverted to form a compact, spheroidal daughter colony. During the cell divisions and inversion, the parental gelatinous matrix gradually expanded and showed an internal vesicle-like structure that encompassed each of the developing embryos as found in *Yamagishiella* [4], *Eudorina* [5], and *Pleodorina* [6]. However, such vesicles were visible only when stained with methylene blue under a light microscope in *Colemanosphaera* (Additional file: Figure S1B,C). Each protoplast of the newly formed daughter colony developed two flagella and subsequently released from the parental colony. In *C. charkowiensis*, flagellar elongation was unequal to produce apparently uniflagellate cells in newly formed daughter colonies (Additional file: Figure S1D), as in *Yamagishiella* and *Eudorina* [1,2,4]. However, two flagella of each protoplast in the newly formed daughter colony grew equally in *C. angeleri* (Additional file: Figure S1E).

Transmission electron microscopy

Sections of the extracellular matrix of vegetative colonies of the two new species of *Colemanosphaera* revealed the tripartite boundary that surrounded the entire colony (Additional file: Figure S2). This colonial boundary is characteristic of the Volvocaceae [7]. Each cell was tightly enclosed by a fibrillar layer of the matrix (cellular envelope) inside the colonial boundary. In *C. charkowiensis*, the cellular envelope was dense under the transmission electron microscopy (Additional file: Figure S2B). However, that of *C. angeleri* was thin (Additional file: Figure S2D) and sometimes indistinguishable from other fibrillar material of the extracellular matrix inside the colonial boundary. A large cup-shaped chloroplast occupied most of the cell periphery and a nucleus was located in the center of the cell (Additional file: Figure S2A-C). Golgi bodies and mitochondrial profiles were detected within the cytoplasm. The pyrenoid was observed within the chloroplast and surrounded by several starch grains. Thylakoid lamellae penetrated into the

pyrenoid matrix through spaces between starch grains as they became tubular.

Molecular phylogenetic analyses

Based on the 5,523 bp in the five chloroplast genes from 58 operational taxonomic units, four clades were resolved with moderate to high support values (0.98-1.00 posterior probability [PP] based on Bayesian inference [BI] and 63-100% bootstrap values in maximum likelihood [ML] and maximum parsimony [MP] methods) within the Volvocaceae: *Eudorina* group, *Yamagishiella*, *Pandorina*-*Volvulina* clade, and a clade (VPC clade) composed of *Volvox* sect. *Volvox*, *Platydorina*, and *Colemanosphaera* (Figure 2). However, phylogenetic relationships among these four clades were not well-resolved. *Eudorina* group was composed of *Eudorina*, *Pleodorina*, and *Volvox* species (excluding *Volvox* sect. *Volvox*) [3, 8] and was sister to *Yamagishiella* with only 0.98 PP. The monophyly of the *Pandorina*-*Volvulina* lineage was supported with high support values (with 1.00 PP based on BI and 95-96% bootstrap values based on ML and MP methods). The VPC clade was supported with moderate support values (with 0.98 PP based on BI and 63-70% bootstrap values based on ML and MP methods). Within the VPC clade, the genus *Colemanosphaera* was sister to *Platydorina* with highest support values (with 1.00 PP based on BI and 100% bootstrap values based on ML and MP calculations).

Phylogenetic relationships resolved based on the ITS region of nuclear *r*DNA (ITS-1, 5.8S *r*DNA and ITS-2) demonstrated that *Colemanosphaera* plus strain ASW05157 were subdivided into two separate clades with 97-100% bootstrap values using the MP and ML methods (Figure 4). One was composed of strains *C. charkowiensis* originating from Japan, whereas the other included a Japanese strain of *C. angeleri* and strain ASW05157 (originating from Regelsbrunn, Austria [9]). Within the aligned region (580 nucleotides) of the ITS region, only a single nucleotide in ITS-1was different between *C. angeleri* and strain ASW05157.

Secondary structures of ITS-2 *r*DNA transcripts

The present phylogenetic analyses resolved that the two new species of *Colemanosphaera* and strain ASW05157 are closely related, namely, sister taxa (Figures 2 and 4). Thus, base changes in ITS-2 *r*DNA were examined among these algae. No CBC was found between *C. angeleri* and strain ASW05157. However, two CBCs were detected in helix I and helix III between *C. angeleri* (including ASW05157) and *C. charkowiensis* (Additional file: Figure S4). The secondary structures of ITS-2 *r*DNA from *Colemanosphaera* and *P. caudata* are shown (Additional file 1: Figures S5-S7).

Table S1. List of strains of *Colemanosphaera* used in this study.

Species	Strain designation [Accession number of ITS region of nuclear ribosomal DNA]	Origin of strain
C. charkowiensis	Isa 7-1 (= NIES[a] -3383) [AB905583]	Water sample collected from Lake Isanuma (water temperature 29.8°C; pH 8.2；N 35° 55' 19", E 139° 30' 55"), Isanuma, Kawagoe-shi, Saitama in 24 June 2005
	2010-0713-E2 (= NIES-3384) [AB905580], 2010-0713-E5 (= NIES-3385) [AB905581]	Water sample collected from Lake Isanuma (water temperature 26.2°C; pH 7.5；N 35° 55' 19", E 139° 30' 55"), Isanuma, Kawagoe-shi, Saitama in 12 July 2010.
	2013-0615-IC-3 (= NIES-3386) [AB905582], 2013-0615-IC-4 (= NIES-3387) [AB905584], 2013-0615-IC-7 (= NIES-3388) [AB905585]	Water sample collected from a small pond (water temperature 27.0°C; pH 6.3; N 35° 55' 17", E 139° 30' 52") just outside Lake Isanuma, Isanuma, Kawagoe-shi, Saitama in 15 June 2013
C. angeleri	2010-0126-1 (= NIES-3382) [AB905586]	Soil sample collected from Lake Isanuma (N 35° 55' 17", E 139° 30' 57"), Isanuma, Kawagoe-shi, Saitama in 20 January 2010

[a] Microbial Culture Collection at the Institute for National Environmental Studies (http://mcc.nies.go.jp/) [10]

Table S2. List of the colonial volvocine taxa/strains included in the phylogenetic analysis (Figure 2) and DDBJ/EMBL/GenBank accession numbers of the five chloroplast genes.

Taxon	Strain designation	Accession no				
		atpB	*rbcL*	*psaA*	*psaB*	*psbC*
Tetrabaenaceae						
Tetrabaena socialis	NIES[a]-571	AB014014	D63443	AB014015	AB044466	AB044525
Basichlamys sacculifera	NIES-566	AB014015	D63430	AB014016	AB044467<br>AB044468	AB044526
Goniaceae						
Gonium pectorale	NIES-569	AB014016<br>AB014017	D63437	AB044242	AB044463	AB044521
Gonium octonarium	NIES-851	AB014018	D63436	AB044241	AB044462	AB044520
Gonium quadratum	NIES-653	AB014019	D63438	AB044243	AB044464	AB044522<br>AB044523
Gonium multicoccum	UTEX[b] 2580	AB014020	D63435	AB044239<br>AB044240	AB044461	AB044481
Gonium multicoccum	UTEX 783	AB076115<br>AB076116	AB076102<br>AB076103	AB076102	AB076153<br>AB076154	AB076168<br>AB076169<br>AB076170<br>AB076171
Gonium viridistellatum	UTEX 2519	AB014021	D86831	AB044244	AB044465	AB044524
Gonium viridistellatum	NIES-857	AB076117	AB076092<br>AB076093	AB076139	AB076155	AB076172
Gonium viridistellatum	NIES-289	AB076118<br>AB076119	AB076091	AB076140<br>AB076141	AB076156	AB076173
Volvocaceae						
Pandorina morum	NIES-574	AB014025<br>AB014036	D63442	AB044226	AB044452	AB044505
Pandorina morum	UTEX 854	AB044180	AB044167	AB044231	AB044456	AB044511<br>AB044510
Pandorina morum	UTEX 880	AB044179	AB044166	AB044229<br>AB044230	AB044455	AB044509
Pandorina morum	UTEX 1727	AB044178	AB044165	AB04428	AB044454	AB044508
Pandorina morum	UTEX 2326	AB044177	AB044164	AB044227	AB044453	AB044506<br>AB044507
Pandorina colemaniae	NIES-572	AB014027	D63441	AB044232	AB044457	AB044512
Volvulina pringsheimii	UTEX 1020	AB014028	D63444d	AB044220	AB044447	AB044499
Volvulina steinii	UTEX 1525	AB044174	AB044160	AB044223	AB044449	AB044501
Volvulina steinii	UTEX 1531	AB044175	AB044161	AB044224	AB044450	AB044502<br>AB044503
Volvulina steinii	NIES-545	AB044173	AB044159	AB044221<br>AB044222	AB044448	AB044500
Volvulina boldii	UTEX 2185	AB044176	AB044162<br>AB044163	AB044225	AB044451	AB044504
Volvulina compacta	NIES-582	AB014029	D86832	AB044217<br>AB044218<br>AB044219	AB044446	AB044498
Yamagishiella unicocca	UTEX 2428	AB014030	D86823	AB044213	AB044443	AB044495
Yamagishiella unicocca	UTEX 2430	AB014031	D86825f	AB044214<br>AB044215	AB044444	AB044496
Yamagishiella unicocca	NIES-872	AB044172	AB044168	AB044216	AB044445	AB044497

Platydorina caudata	UTEX 1658	AB014032	D86828	AB044211<br>AB044212	AB044442	AB044494
Colemanosphaera charkowiensis	Isa 7-1<br>(=<br>NIES-3383)	AB905589	AB905591	AB905593	AB905595	AB905597
Colemanosphaera angeleri	2010-0126-1<br>(=<br>NIES-3382)	AB905590	AB905592	AB905594	AB9055956	AB905598
Eudorina cylindrica	UTEX 1197	AB014033	D86833	AB044210	AB044441	AB044493
Eudorina peripheralis	UTEX 1215[c]	AB014007	D63434	AB044207<br>AB044208<br>AB044209	AB044440	AB044491<br>AB044492
Eudorina unicocca	UTEX 737	AB014008	D86829	AB044204<br>AB044205<br>AB044206	AB044439	AB044489<br>AB044490
Eudorina elegans	NIES-456	AB014009	D63432	AB044199	AB044435	AB044485
Eudorina elegans	UTEX 1205	AB014010	D88805	AB044200<br>AB044201	AB044436<br>AB044437	AB044486
Eudorina elegans	UTEX 1212	AB014012	D88806	AB044202<br>AB044203	AB044438	AB044487<br>AB044488
Eudorina illinoisensis	NIES-460	AB014013	D63433	AB0440198	AB044434	AB044484
Pleodorina thompsonii	UTEX 2804	AB214407	AB214408	AB214410<br>AB214411	AB214412	AB214413
Pleodorina starrii	NIES-1362	AB214424	AB214427	AB214430	AB214432	AB214434
Pleodorina indica	UTEX 1990	AB014006	D86834	AB044195<br>AB044196<br>AB044197	AB044432<br>AB044433	AB044483
Pleodorina japonica	UTEX 2523	AB014005	D63440	AB044193<br>AB044194	AB044431	AB044482
Pleodorina californica	UTEX 809	AB014004	D63439	AB044190<br>AB044191<br>AB044192	AB044430	AB044480
Volvox gigas	UTEX 1895	AB076112	AB076084	AB076131<br>AB076132	AB076150	AB076165
Volvox ovalis	NIES-2569	AB592341	AB592342	AB592339	AB592340	AB592338
Volvox obversus	UTEX 1865	AB076113	AB076085	AB076133<br>AB076134<br>AB076135<br>AB076136	AB076151	AB076166
Volvox africanus	UTEX 1891	AB076114	AB076101	AB076137	AB076152	AB076167
Volvox tertius	UTEX 132	AB076106<br>AB076107	AB076098	AB076125<br>AB076126	AB076147	AB076162
Volvox tertius	NIES-544	AB086173	AB086174	AB086175<br>AB086176	AB086177	AB086178
Volvox powersii	UTEX 1863	AB214414	AB214415	AB214416	AB214417	AB214418
Volvox carteri						
f. *kawasakiensis*	NIES-732	AB013999	D63446	AB044184<br>AB044185	AB044425	AB044475
f. *nagariensis*	UTEX 1885	AB076108<br>AB075109	AB076099	AB076127<br>AB076128	AB076148	AB076163
f. *weismannia*	UTEX 1875	AB076110<br>AB076111	AB076100	AB076129<br>AB076130	AB076149	AB076164
Volvox aureus	NIES-541	AB013998	D63445	AB044182	AB044424	AB04447
Volvox aureus	NIES-891	AB076104	AB076096	AB076123	AB076145	AB076160
Volvox aureus	NIES-892	AB076105	AB076086	AB076124	AB076146	AB076161

Volvox dissipatrix	UTEX 2184	AB014000	D63447	AB044183	AB044426	AB044476
Volvox dissipatrix	Marb.2RS 29	AB214419	AB214420	AB214421	AB214422	AB214423
Volvox rousseletii	UTEX 1862	AB014003	D63448	AB044188	AB044429	AB044479
Volvox barberi	UTEX 804	AB014001	D86835	AB044186	AB044427	AB044477
Volvox globator	UTEX 955	AB014002	D86836	AB044187	AB044428	AB044478

[a] Microbial Culture Collection at the Institute for National Environmental Studies (http://mcc.nies.go.jp/) [10]

[b] Culture Collection of Algae at the University of Texas at Austin (http://web.biosci.utexas.edu/utex/default.aspx) [11]

[c] Re-identified by Yamada et al. [12]

References

1. Goldstein M**: Speciation and mating behaviour in *Eudorina*.** *J Protozool* 1964, **11**:317-344.
2. Nozaki H, Ito M: **Phylogenetic relationships within the colonial Volvocales (Chlorophyta) inferred from cladistic analysis based on morphological data.** *J Phycol* 1994, **30**:353–365.
3. Nozaki H, Ott FD, Coleman AW: **Morphology, molecular phylogeny and taxonomy of two new species of *Pleodorina* (Volvoceae, Chlorophyceae)**. *J Phycol* 2006, **42**:1072-1080.
4. Nozaki H: T**he life history of Japanese *Pandorina unicocca* (Chlorophyta, Volvocales).** *J Jpn Bot* 1981, **56**: 65-72, pls. 1-2.
5. Nozaki H, Krienitz L: **Morphology and phylogeny of *Eudorina minodii* (Chodat) Nozaki et Krienitz, comb. nov. (Volvocales, Chlorophyta) from Germany.** *Eur J Phycol* 2001, **36**: 23-8.
6. Nozaki H, Kuroiwa H, Mita T, Kuroiwa T: ***Pleodorina japonica* sp. nov. (Volvocales, Chlorophyta) with bacteria-like endosymbionts**. *Phycologia* 1989, 28: 252-267.
7. Nozaki H, Kuroiwa T: **Ultrastructure of the extracellular matrix and taxonomy of *Eudorina*, *Pleodorina* and *Yamagishiella* gen. nov. (Volvocaceae, Chlorophyta).** *Phycologia* 1992, 31: 529-541.
8. Nozaki H, Misawa K, Kajita T, Kato M, Nohara S, Watanabe MM: **Origin and evolution of the colonial Volvocales (Chlorophyceae) as inferred from multiple, chloroplast gene sequences.** *Mol Phylogenet Evol* 2000, **17**: 256–268.
9. Angeler DG, Schager M, Coleman AW: **Phylogenetic relationships among isolates of *Eudorina* species (Volvocales, Chlorophyta) inferred from molecular and biochemical data.** *J Phycol* 2002, **35**: 815-823.
10. Kasai F, Kawachi M, Erata M, Mori F, Yumoto K, Sato M, Ishimoto M (Eds): **NIES-Collection. List of Strains. 8th Edition.** *Jpn J Phycol* 2009, **57** (1), Supplement: 1-350, pls. 1-7.
11. Starr RC, Zeikus JA: **UTEX--The Culture Collection of Algae at The University of Texas at Austin.** *J Phycol* 1993, **29** (suppl.):1-106.
12. Yamada TK, Miyaji K, Nozaki H: **A taxonomic study of *Eudorina unicocca* (Volvocaceae, Chlorophyceae) and related species, based on morphology and molecular phylogeny.** *Eur J Phycol* 2008, **43**:317-326.
